# Association between loss of hypercoagulable phenotype, clinical features and complement pathway consumption in COVID-19

**DOI:** 10.3389/fimmu.2024.1337070

**Published:** 2024-03-11

**Authors:** Daisuke Kasugai, Taku Tanaka, Takako Suzuki, Yoshinori Ito, Kazuki Nishida, Masayuki Ozaki, Takeo Kutsuna, Toshiki Yokoyama, Hitoshi Kaneko, Ryo Ogata, Ryohei Matsui, Takahiro Goshima, Hiroshi Hamada, Azusa Ishii, Yusuke Kodama, Naruhiro Jingushi, Ken Ishikura, Ryo Kamidani, Masashi Tada, Hideshi Okada, Takanori Yamamoto, Yukari Goto

**Affiliations:** ^1^ Department of Emergency and Critical Care Medicine, Nagoya University Graduate School of Medicine, Nagoya, Japan; ^2^ Department of Pediatrics, Nagoya University Graduate School of Medicine, Nagoya, Japan; ^3^ Department of Biostatistics, Nagoya University Graduate School of Medicine, Nagoya, Japan; ^4^ Department of Critical Care Medicine, Komaki City Hospital, Komaki, Japan; ^5^ Department of Respiratory Medicine, Daido Hospital, Nagoya, Japan; ^6^ Department of Emergency and Critical Care Medicine, Tosei General Hospital, Seto, Japan; ^7^ Department of Emergency and Critical Care Medicine, Tokyo Metropolitan Tama Medical Center, Fuchu, Japan; ^8^ Department of Respiratory Medicine, Meitetsu Hospital, Nagoya, Japan; ^9^ Department of Emergency and Critical Care Medicine, Nagoya City University Hospital, Nagoya, Japan; ^10^ Department of Emergency and General Internal Medicine, Fujita Health University, Toyoake, Japan; ^11^ Department of Internal Medicine, National Hospital Organization Nagoya Medical Center, Nagoya, Japan; ^12^ Department of Respiratory Medicine, Chukyo Hospital, Nagoya, Japan; ^13^ Department of Internal Medicine, Kyoritsu General Hospital, Nagoya, Japan; ^14^ Department of Emergency and Disaster Medicine, Mie University Graduate School of Medicine, Tsu, Japan; ^15^ Department of Emergency and Critical Care Medicine, Gifu University Graduate School of Medicine, Gifu, Japan; ^16^ Department of Internal Medicine, SaiShukan Hospital, Kitanagoya, Japan; ^17^ Department of Emergency Medicine, Nagoya EkiSaikai Hospital, Nagoya, Japan

**Keywords:** COVID-19, blood coagulation disorders, rotational thromboelastometry, alternative complement pathway, microthrombosis

## Abstract

**Background:**

Coronavirus disease 2019 (COVID-19) features a hypercoagulable state, but therapeutic anticoagulation effectiveness varies with disease severity. We aimed to evaluate the dynamics of the coagulation profile and its association with COVID-19 severity, outcomes, and biomarker trajectories.

**Methods:**

This multicenter, prospective, observational study included patients with COVID-19 requiring respiratory support. Rotational thromboelastometry findings were evaluated for coagulation and fibrinolysis status. Hypercoagulable status was defined as supranormal range of maximum clot elasticity in an external pathway. Longitudinal laboratory parameters were collected to characterize the coagulation phenotype.

**Results:**

Of 166 patients, 90 (54%) were severely ill at inclusion (invasive mechanical ventilation, 84; extracorporeal membrane oxygenation, 6). Higher maximum elasticity (*P*=0.02) and lower maximum lysis in the external pathway (*P*=0.03) were observed in severely ill patients compared with the corresponding values in patients on non-invasive oxygen supplementation. Hypercoagulability components correlated with platelet and fibrinogen levels. Hypercoagulable phenotype was associated with favorable outcomes in severely ill patients, while normocoagulable phenotype was not (median time to recovery, 15 days vs. 27 days, *P*=0.002), but no significant association was observed in moderately ill patients. In patients with severe COVID-19, lower initial C3, minimum C3, CH50, and greater changes in CH50 were associated with the normocoagulable phenotype. Changes in complement components correlated with dynamics of coagulation markers, hematocrit, and alveolar injury markers.

**Conclusions:**

While hypercoagulable states become more evident with increasing severity of respiratory disease in patients with COVID-19, normocoagulable phenotype is associated with triggered by alternative pathway activation and poor outcomes.

## Introduction

Coronavirus disease 2019 (COVID-19) triggered a global pandemic by causing primarily, respiratory distress occasionally escalating into respiratory failure, thereby posing significant public health challenges worldwide (1). In this context, coagulopathy, characterized by hypercoagulation is identified as one of the key pathogenic factors in COVID-19 (2, 3). Associations of COVID-19 with macro- and microthrombosis have been established (4–6), which have spurred numerous clinical trials aiming to mitigate coagulopathy induced by this disease (7). Despite these efforts, previous studies did not consistently demonstrate the efficacy of therapeutic anticoagulation against COVID-19, irrespective of disease severity (8). While the effectiveness of therapeutic anticoagulation has been explored in moderately and critically ill patients with COVID-19 (9, 10), the contrasting results from these trials suggest that therapeutic anticoagulation might be more effective in non-critically ill patients than in their critically ill counterparts. This discrepancy could stem from the evolving nature of coagulopathy as the disease progresses. Hence, understanding how coagulopathy varies with disease severity is essential for optimizing therapeutic interventions aimed at controlling coagulation status.

This study aimed to investigate the changes in coagulation characteristics with increasing severity of COVID-19.

## Methods

### Study population and setting

This prospective observational study conducted at 14 centers in Japan, from March 2021 to March 2022 included individuals aged ≥18 years, who were hospitalized for COVID-19 pneumonia and required oxygenation. COVID-19 was diagnosed based on findings from lung imaging and a positive severe acute respiratory syndrome coronavirus 2 (SARS-CoV2) polymerase chain reaction or antigen test. Patients were classified into four groups based on COVID-19 severity at the time of coagulation status assessment according to the WHO ordinal scale: moderately ill patients requiring supplemental oxygen, patients on non-invasive respiratory support, patients on mechanical ventilation, and patients on extracorporeal membrane oxygenation (11). Exclusion criteria included known coagulopathy, previous oral anticoagulants administration, hematologic malignancy, and thrombocytopenia (detailed criteria listed in the **Supplementary Methods** in **Additional File 1**). Patients provided pre-enrollment informed consent. The study was approved by Nagoya University Hospital Institutional Review Board (Approval No. 2020-0548).

### Evaluation of coagulation profile using thromboelastometry

**Figure 1:**
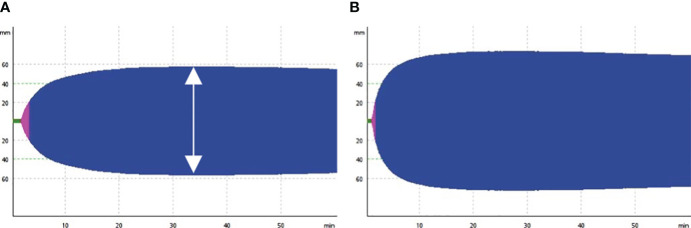
Representative image of thromboelastometry test. The figure shows the output of the EXTEM component of thromboelastometry, illustrating normal coagulation **(A)** and hypercoagulable states in patient with COVID-19 patients **(B)**. The double arrow indicates maximum clot firmness.

In addition to clinical laboratory coagulation function tests, thromboelastometry with ROTEM sigma™ was used to evaluate the coagulation profile of participants on the day of inclusion. The maximum clot elasticity (MCE) of each component was calculated from maximum clot firmness (MCF) using the previously described: MCE = (100×MCF)/(100-MCF) (12). The platelet component of MCE is evaluated using the difference between MCE EXTEM and FIBTEM. A hypercoagulable state was defined as the MCE of the external pathway exceeding the normal reference value (13). **Figure 1** illustrates responentable image of EXTEM components of the thromboelastometry test for patients with normal and hypercoagulable states. A comprehensive description of the thromboelastometry parameters can be found in the previous publicaion (14).

### Outcomes and clinical data

The primary endpoint was the time to recovery, with the recovery day defined as the first day during the 28 days after enrollment, on which patients did not require any respiratory support (11). Secondary outcomes included ventilator-free days within the 28 days and in-hospital mortality. Other recorded clinical data included demographics, comorbidity, activities of daily living (15), history of COVID-19 vaccinations, respiratory mechanics, laboratory values at the time of thromboelastometry evaluation, and treatments. In patients requiring mechanical ventilation at the Nagoya University Hospital, daily laboratory results within the initial 7 days from intubation to ICU discharge were recorded to assess the association between biomarker dynamics. The collected longitudinal data were analyzed to identify the initial, the highest, and lowest value, and calculate the maximum changes(ΔC3 = lowest C3/initial C3 ×100%). These routine lab tests were conducted by the central laboratory, with the specific testing methods and reagents detailed in **Table E1** in the **Supplementary Material**.

### Viral load assessment and genotyping of SARS-CoV-2

To explore the relationship with coagulation profiles, plasma viral loads were analyzed using quantitative reverse-transcription polymerase chain reaction. To investigate the relationship between coagulation profiles and viral genotypes in SARS-CoV-2, we performed whole-genome sequencing using stored specimens from the lower respiratory tract.

### Statistical analysis

Spearman’s rank correlation test was used for statistical analysis, focusing on assessing the relationships between thromboelastometry parameters and clinical laboratory tests, such as coagulation function tests, using Spearman’s rank correlation test. The results were visualized using a heatmap. This method was also employed to examine longitudinal associations between complements and laboratory biomarkers. To assess differences in thromboelastometry results by severity, the Kruskal–Wallis test was used. *P* values for pairwise comparisons were adjusted using the Bonferroni method. The association between hypercoagulable state and baseline respiratory and laboratory parameters was evaluated using the Mann–Whitney U test. To decrease type-I error of multiple comparisons for 29 variables, the Benjamini–Hochberg procedure with a false discovery rate of 0.05 was applied to adjust the *P* value. Time to recovery was evaluated using the log-rank test, with a competing event of death regarded as “not recovered” on the last observation day, following an approach similar to the Fine–Grey method (11, 16). A *P* value less than 0.05 was considered statistically significant. All statistical analyses were performed using R software (version 4.2.2) and RStudio.

## Results

### Characteristics of patients and outcomes

Of the 192 cases identified during the observation period, 166 were finally included in the study (**Figure E1** in the **Additional File 1**). Among the enrolled patients, 76 had moderate respiratory failure at the time of inclusion (conventional oxygen supplementation, 62; non-invasive respiratory support, 14), and 90 had severe respiratory failure (mechanical ventilation, 84; extracorporeal membrane oxygenation, 6) (**Table 1**). The most common comorbidities were hypertension (40%) and diabetes mellitus (28%). Prior to disease onset, 159 (96%) patients could independently perform activities of daily living. Of the enrolled patients, 26 (16%) had received two doses of SARS-CoV-2 vaccine before disease onset. Unfractionated heparin was more commonly administered in severe cases, 88 (98%), than in moderate ones, 34 (45%), and was mainly used for therapeutic dose titration. Among patients with moderate respiratory failure at the time of inclusion, 10 (13%) experienced disease progression. Of the patients who received mechanical ventilation, 59 (66%) recovered after 28 days, 11 (12%) died, and 20 (22%) became ventilator dependent.

**Table 1:** Baseline characteristics of study participants.

Characteristic	Moderate, N = 76^1^	Severe, N = 90^1^	p-value^2^
Respiratory support			<0.001
ECMO	0 (0%)	6 (6.7%)	
Invasive Mechanical Ventilation	0 (0%)	84 (93%)	
Non-Invasive Respiratory Support	14 (18%)	0 (0%)	
Supplemental Oxygen	62 (82%)	0 (0%)	
Age (years)	56 (49, 66)	58 (53, 68)	0.2
Male sex	58 (76%)	72 (80%)	0.6
Body weight (kg)	72 (60, 81)	72 (64, 85)	0.7
Height (cm)	165 (160, 173)	170 (160, 175)	0.5
ADL			0.3
Independent	71 (93%)	88 (98%)	
Partially dependent	4 (5.3%)	1 (1.1%)	
Completely dependent	1 (1.3%)	1 (1.1%)	
Comorbidity			
Alcohol abuse	2 (2.6%)	0 (0%)	0.2
Diabetes Mellitus	18 (24%)	29 (32%)	0.2
Hypertension	26 (34%)	41 (46%)	0.14
Chemotherapy within 30 days	1 (1.3%)	1 (1.1%)	>0.9
Heart failure	3 (3.9%)	4 (4.4%)	>0.9
Chronic liver disease	1 (1.3%)	2 (2.2%)	>0.9
Chronic lung disease	7 (9.2%)	6 (6.7%)	0.5
End-stage renal disease	11 (14%)	15 (17%)	0.7
Prior COVID- 19 vaccination	11 (14%)	15 (17%)	0.7
Antiplatelet medication	8 (11%)	9 (10%)	>0.9
Anticoagulation medication			
Unfractionated heparin	34 (45%)	88 (98%)	<0.001
Therapeutic anticoagulation	17 (22%)	71 (79%)	<0.001
Anti-viral medication			
Remdesivir	75 (99%)	78 (87%)	0.004
Favipiravir	1 (1.3%)	6 (6.7%)	0.13
Anti-inflammatory drug			
Baricitinib	29 (38%)	43 (48%)	0.2
Tocilizumab	31 (41%)	16 (18%)	0.001
Dexamethasone (6.6 mg/day)	60 (79%)	75 (83%)	0.5
Steroid pulse (mPSL 1000 mg/day)	37 (49%)	62 (69%)	0.008
Recovery at 28 days	70 (92%)	59 (66%)	<0.001
In-hospital death	3 (3.9%)	11 (12%)	0.056

^1^n (%); Median (IQR).

^2^Fisher’s exact test; Wilcoxon rank sum test; Pearson’s Chi-squared test.

### Coagulation profiles vary according to the severity of respiratory failure

**Figure 2:**
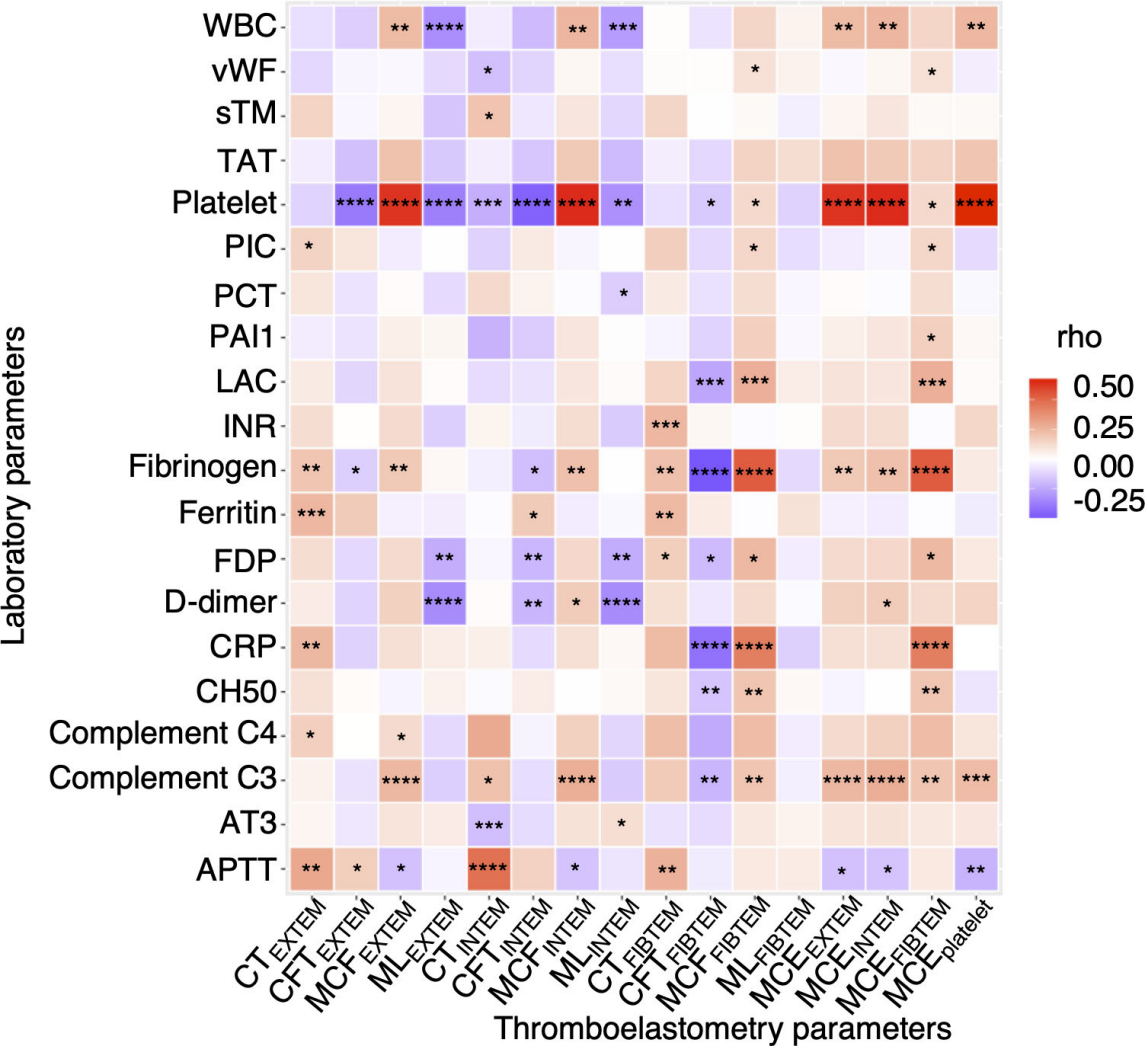
Correlation of thromboelastometry results with clinical laboratory test results. A heatmap showing correlation of viscoelasticity test parameters with clinical laboratory test results. Each rectangle represents Spearman’s rank correlation coefficient. *P* values are displayed in asterisks. * *P* value < 0.05, ** *P* value < 0.01, *** *P* value < 0.001, ****P value < 0.0001. WBC, white blood cell; vWF, von Willebrand factor; sTM, soluble thrombomodulin; TAT, thrombin-antithrombin complex; PIC, plasmin-alpha 2-plasmin inhibitor complex; PCT, procalcitonin; PAI1, plasminogen activator inhibitor-1; LAC, lupus anticoagulant; INR, international normalized ratio; FDP, fibrin degradation product; CRP, c reactive protein; CH50, total hemolytic complement; AT3, antithrombin 3; APTT, activated partial thrombin time; CT, clotting time; CFT, clot formation time; MCF, maximum clot firmness; ML, maximum lysis; MCE, maximum clot elasticity.

**Figure 3:**
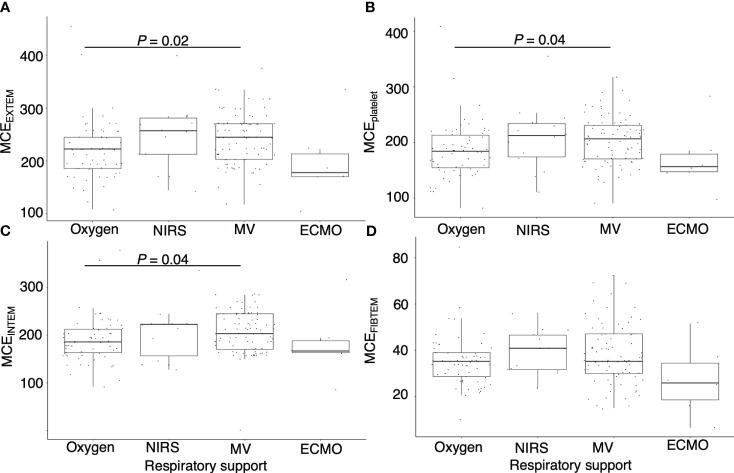
Association between maximum clot elasticity and severity of respiratory distress. Box plots showing **(A)** maximum clot elasticity of EXTEM for each severity level, **(B)** maximum clot elasticity of platelet components for each severity level, **(C)** maximum clot elasticity of INTEM for each severity level, and **(D)** maximum clot elasticity of FIBTEM for each severity level. MCE, maximum clot elasticity; NIRS= non-invasive respiratory support; MV, mechanical ventilation; ECMO, extracorporeal membrane oxygenation. *P* values calculated from the Mann–Whitney U test for pairwise comparison and adjusted using the Bonferroni method. Adjusted *P* values are displayed only if <0.05.

**Figure 4:**
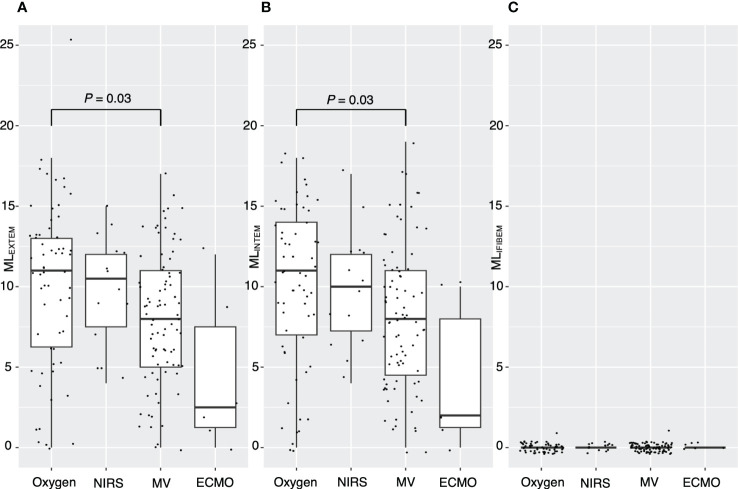
Association between maximum lysis and severity of respiratory distress. Box plots showing **(A)** maximum lysis of EXTEM for each severity level, **(B)** maximum lysis of INTEM for each severity level, and **(C)** maximum lysis of FIBTEM for each severity level. ML, maximum lysis; NIRS, non-invasive respiratory support; MV, mechanical ventilation; ECMO, extracorporeal membrane oxygenation. *P* values calculated from the Mann–Whitney U test for pairwise comparison and adjusted using the Bonferroni method. *P* values are displayed if *P <*0.05.

**Figure 2** demonstrates a correlation between thromboelastometry parameters and laboratory biomarkers, where MCF and MCE were positively correlated with platelet counts (rho for MCE_EXTEM_ = 0.67, *P* < 0.001) in all components except for in FIBTEM. Maximum lysis parameters were weak and negatively associated with white blood cell count, D-dimer levels, and platelet count (rho = -0.3, -0.32, and -0.32, respectively, for maximum lysis (EXTEM), *P* < 0.001). **Figures 3**, **4** illustrate the relationship between elasticity and fibrinolytic profile among different severities of respiratory failure. Patients receiving non-invasive respiratory support exhibited the highest MCE_EXTEM_ values among other severity levels (**Figure 3A**, median [interquartile range (IQR)], 223 [186-245] for supplemental oxygen, 257 [213-281] for on-invasive respiratory support, 244 [203-270] for mechanical ventilation, and 178 [170-213] for extracorporeal membrane oxygenation, respectively).MCE was higher in patients on mechanical ventilation than in those receiving supplemental oxygen, which mainly reflects the platelet component (**Figure 3B**, median [IQR] MCE_platelet_ of supplemental oxygen vs mechanical ventilation, 184 [154–213] vs. 206 [170–230], *P* = 0.02). Meanwhile, maximum lysis declined as respiratory failure progressed (**Figures 4A, B**, median [IQR] ML_EXTEM_ of supplemental oxygen vs mechanical ventilation, 11 [6–13] vs. 8 [5–11], *P* = 0.03). No significant differences between the groups were observed in terms of the fibrinogen component (**Figures 3D**, **4C**).

### Hypercoagulable state in patients with severe COVID-19 is associated with favorable prognosis

**Figure 5:**
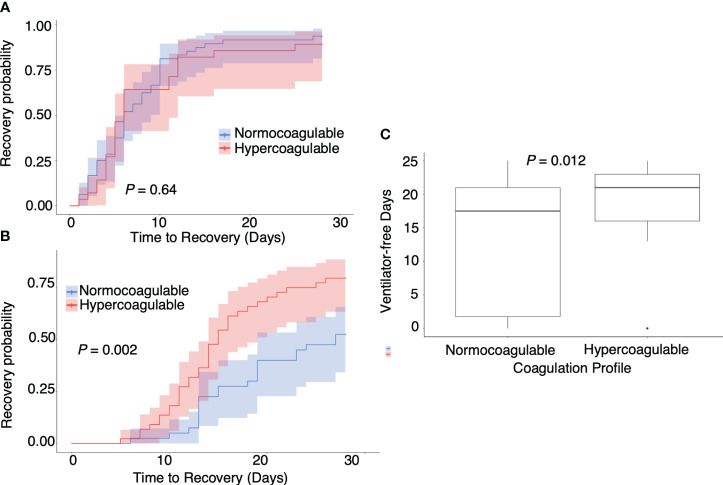
Association between coagulation profile and outcomes. Association between **(A)** coagulation profile and time to recovery in moderate COVID-19 cases, **(B)** coagulation profile and time to recovery in severe COVID-19 cases, and **(C)** coagulation profile and ventilator-free days in severe COVID-19 cases. *P* values calculated from the Mann–Whitney U test for pairwise comparison and adjusted using the Bonferroni method. *P* values are displayed if *P <*0.05.

**Figure 6:**
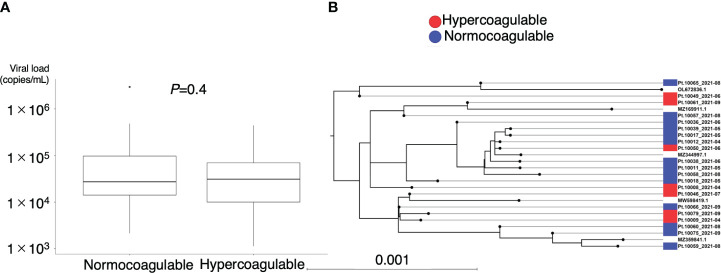
Association between coagulation profile and viral load and genotype. **(A)** Plasma viral load of SARS-CoV-2 at the time of coagulation profile evaluation. **(B)** Phylogenetic analysis of SARS-CoV-2 sequences obtained from the patients (n = 21) and their coagulation phenotypes. Scale bar indicates the measure of evolutionary distance calculated from the proportion of differing bases in the total sequence. Representative strains are indicated by GenBank accession number as references. OL672836.1(Omicron BA.1); MZ169911.1(Gamma P.1); MZ344997.1(Alpha B.1.1.7); MW598419.1(Beta B.1.351); MZ359841.1(Delta B.1.617.2).

**Figure 5** illustrates the association between hypercoagulable status and clinical outcomes. No correlation was observed between hypercoagulable state and the disease course in moderate COVID-19 cases (**Figure 5A**, median [95% confidence interval (CI)] time to recovery of hypercoagulable group vs. normocoagulable group, 6 [5–11] days vs. 6 [5–9] days, *P* = 0.66). However, in severe COVID-19 cases, hypercoagulable state was associated with a favorable prognosis (**Figures 5B, C**, median [95% CI] time to recovery, 15 [13–18] days vs. 27 [19–censored] days, *P* = 0.002; median [IQR] ventilator-free days, 18 [2–21] vs. 21 [16–23], *P* = 0.01). There was no significant difference in mortality associated with the two coagulation states (15% vs. 13%). The normocoagulable state in severe COVID-19 cases was associated with lower fibrinogen (median [IQR], 551 [481–579] mg/dL vs. 428 [359–534] mg/dL, adjusted *P* value = 0.03), lower platelet count (median [IQR], 246 [194–308] × 10^3^/μL vs. 154 [126–204] × 10^3^/μL, adjusted *P* value <0.001), and lower complement C3 value (median [IQR], 116 [100–128] mg/dL vs. 96 [73–118] mg/dL, adjusted *P* value = 0.05) (**Table E3** in **Additional File 1**). No significant differences in respiratory parameters were observed between the two coagulation states. Further analysis in severe COVID-19 cases demonstrated no correlation between viral load and hypercoagulable state; there was no clear association between the viral genotype and hypercoagulable state (**Figure 6**).

### Association between coagulation profile and complement dynamics

**Figure 7:**
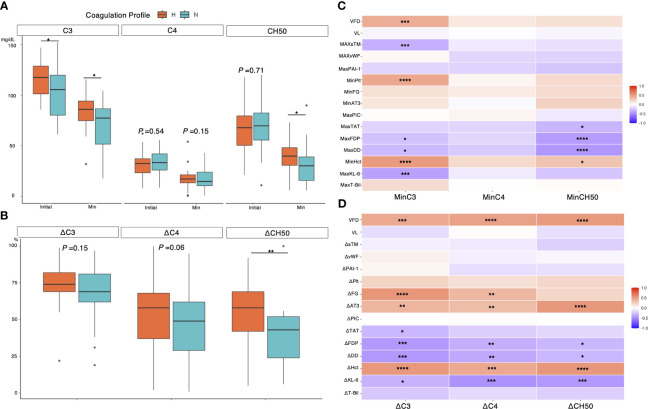
Association between complement dynamic and biomarkers in severe COVID-19 cases. **(A)** Association between coagulation profiles and initial minimum value for each complement component. **(B)** Association between coagulation profiles and changes in each complement component. **(C)** A heatmap showing association between minimum complement value and biomarkers, clinical outcome, and viral load evaluated with Spearman’s rank correlation coefficient. **(D)** A heatmap showing association between changes for each complement component and variables. Each laboratory variable was evaluated daily for 7 days from the day of intubation until ICU discharge. Minimum/Maximum values of each variable within 7 days were recorded and used to calculate the changes (e.g., ΔC3 = minimum C3/initial C3 ×100%). *P* values are displayed in asterisks. * *P* value < 0.05, ** *P* value < 0.01, *** *P* value < 0.001, ****P value < 0.0001. H= hypercoagulable status; N, normocoagulable status; CH50= total complement activity; VFD, ventilator-free days; VL, plasma viral load; sTM, soluble Thrombomodulin; vWF= von Willebrand factor; PAI-1= plasminogen activator inhibitor-1; Plt, platelet count; FG, fibrinogen; AT3, antithrombin 3; PIC, plasmin-alpha 2-plasmin inhibitor complex; TAT, thrombin-antithrombin complex; FDP, fibrin degradation product; DD, D-dimer; Hct, hematocrit; KL-6, Krebs von den lungen-6; T-bil, total bilirubin.

**Figure 7** illustrates the relationship between coagulation profiles and the dynamics of complement components. Complement C3, in addition to the initial assessment, had a significantly lower minimum value in the normocoagulable group (median [IQR], 85.9 [74.8–94.1] mg/dL vs. 77.3 [51.6–86.6] mg/dL, *P* = 0.016) (**Figure 7A**). Meanwhile, any significant difference in the dynamics of C4 was not found between the two coagulation profile groups. Total complement activity was significantly reduced in patients in the normocoagulable group compared to those in the hypercoagulable group (median [IQR] reductions of 58 [42–69]% versus 43 [24–52]%, respectively, P = 0.001) (**Figure 7B**). Decreases in C3 minimum correlated with the decrease in platelet count(rho = 0.56, *P* < 0.001), and hematocrit minimums (rho = 0.61, *P* < 0.001) and were associated with increases in D-dimer levels(rho = -0.56, *P* < 0.001), fibrin degradation product(rho = -0.51, *P* < 0.001), KL-6(rho = -0.45, *P* < 0.001), and soluble thrombomodulin maxima (rho = -0.33, *P* = 0.01) (**Figure 7C**). Notably, changes in each component of complement system (ΔC3, ΔC4, and ΔCH50) were positively correlated with changes in hematocrit (rho = 0.53, 0.46, and 0.53, respectively, *P* < 0.001 for each) and ventilator-free days (rho = 0.45, 0.5, and 0.53, respectively, *P* < 0.001 for each), and negatively correlated with changes in KL-6 (rho = -0.34, -0.48, and -0.45, respectively, *P* = 0.007, <0.001, and <0.001, respectively) (**Figure 7D**).

## Discussion

In this study, we evaluated the coagulation profiles of patients with different COVID-19 severities and found that while hypercoagulation was common with worsening respiratory failure, absence of hypercoagulation was associated with poor prognosis in severely ill patients. Platelet components were mainly involved in changes in blood viscoelasticity. Furthermore, the absence of hypercoagulation was also associated with coagulopathy, with complement consumption initiated by the alternative pathway activation.

Hypercoagulable state is considered a risk for COVID-19 macro-micro thrombosis, and treatment aimed at controlling hypercoagulation has been well studied so far (17). However, the efficacy of therapeutic anticoagulation and antiplatelet therapy has not been illustrated in critically ill patients (18). Our study depicted that hypercoagulable status was associated with rather favorable outcomes, which might be one of the reasons that previous randomized control trials failed to show the efficacy of anticoagulation in critically ill patients with COVID-19 (7–10). The hypercoagulable state is known to be influenced primarily by changes in platelet component (12), and individualized anticoagulation therapies to control the component of hypercoagulability in critically ill patients may be promising (19, 20). We demonstrated that thromboelastometry may be a simple and effective way to stratify severity and to identify different phenotypes of coagulation status. In this study, therapeutic anticoagulation and anti-inflammatory therapy were the standard of care in most critically ill patients. This may have partially led to a favorable outcome in patients with coagulable phenotype compared to the outcome in patients with normocoagulable phenotype. Whether phenotypic differences in coagulation profiles can help in individual treatment selection, such as anticoagulation therapy, needs further evaluation.

Our study showed that complement consumption was associated with normocoagulable phenotype and with poor prognosis in mechanically ventilated patients with COVID-19. Lower C3 but not C4 observed in the normocoagulable group at the initial assessment is consistent with similar results of previous *in vivo* studies that reported that SARS-CoV-2 triggers the alternative pathway overactivation (21). Previous studies reported indirect activation of the alternative pathway in addition to direct activation by viral components (22): viral infection of the alveolar epithelium activates the alternative pathway via the Janus kinase/signal transducer and activator of transcription pathway (23). In our study, elevated KL-6, a biomarker of type II alveolar cells, was associated with decreased C3, and there was no significant association between the viral load and decreased C3. Based on these findings, we speculate that the host response, via type 2 alveolar epithelial cell injury rather than pathogen factors, leads to overactivation of the alternative pathway, which results in phenotypic changes in coagulation phenotype and poor prognosis. The phenotypic difference may be triggered by host heterogeneity such as genetic variants in C3 genes reported in a previous study (24). We also showed that complement consumptions were correlated with reduction in coagulation components. These findings are also consistent with those of a recent randomized trial, which reported that target C3 blockage suppressed thrombin production (25). In contrast to the findings of a previous study (26), we observed that the alternative pathway consumption was associated with consumptive coagulopathy but not with hypercoagulable profile. Higher levels of D-dimer and fibrin degradation product in relation to complement dynamics, together with the findings that maximum lysis decreases as the severity increases, may imply the activity of microthrombosis. Our findings are further supported by ex vivo experiments on virus-induced senescence, which demonstrate that the secretome enhances coagulation, promotes the formation of the lytic complement complex C5b-C9, and stimulates the formation of Neutrophil Extracellular Traps (27, 28). Additionally, a rise in C3a enhances the differentiation of CD16 positive cytotoxic T cells in COVID-19 (29). These underlying processes are implicated in causing lung damage from COVID-19 and result in delayed recovery. The higher maximum soluble thrombomodulin and lower minimum hematocrit may suggest the feature of thrombotic microangiopathy (30, 31). Further studies are warranted to understand the control overactivation of the alternative pathway, especially in high-risk phenotypes.

The strength of this study was the standardized introduction of evidence-based treatments in many patients from multiple hospitals, which was useful in visualizing uncontrolled pathologies in a real-world setting. However, this study had some limitations. First, as the current cohort was a predominantly unvaccinated population, extrapolation to a post-vaccinated population may not be possible. Second, considering that the SARS-CoV-2 outbreak in the region was sporadic and the local health office conducted triage of hospitalized patients during the outbreak, selection bias may have occurred in hospitalized patients. Lastly, the present study is a hypothesis-generating analysis, and further investigation is needed to validate the stratification of severity by coagulation profile and the possibility of individualizing treatment for each phenotype.

In summary, the coagulation profile changes according to the severity of respiratory failure in COVID-19. As per our findings, normocoagulable status characterized by C3 consumption is associated with delayed recovery in patients with severe COVID-19. These findings support the heterogeneous treatment effect of anticoagulation depending on COVID-19 severity. Further studies are warranted to develop phenotype-specific treatments based on the coagulation profiles.

## Data availability statement

The original contributions presented in the study are included in the article/supplementary material, further inquiries can be directed to the corresponding author/s. Nucleotide sequence data reported are available in the DDBJ Sequenced Read Archive under the accession numbers DRX512987-DRX513007.

## Ethics statement

The studies involving humans were approved by Nagoya University Hospital Institutional Review Board. The studies were conducted in accordance with the local legislation and institutional requirements. The participants provided their written informed consent to participate in this study.

## Author contributions

DK: Conceptualization, Data curation, Funding acquisition, Investigation, Methodology, Project administration, Visualization, Writing – original draft, Writing – review & editing. TT: Data curation, Investigation, Supervision, Visualization, Writing – review & editing. TS: Data curation, Investigation, Methodology, Supervision, Writing – review & editing. YI: Data curation, Methodology, Supervision, Writing – review & editing. KN: Formal Analysis, Funding acquisition, Methodology, Supervision, Writing – review & editing. MO: Investigation, Supervision, Writing – review & editing. TK: Data curation, Investigation, Supervision, Writing – review & editing. TYo: Data curation, Investigation, Supervision, Writing – review & editing. HK: Data curation, Investigation, Supervision, Writing – review & editing. RO: Data curation, Investigation, Supervision, Writing – review & editing. RM: Data curation, Investigation, Supervision, Writing – review & editing. TG: Data curation, Investigation, Supervision, Writing – review & editing. HH: Data curation, Investigation, Supervision, Writing – review & editing. AI: Investigation, Supervision, Writing – review & editing, Data curation. YK: Data curation, Investigation, Supervision, Writing – review & editing. NJ: Data curation, Investigation, Supervision, Writing – review & editing. KI: Data curation, Investigation, Supervision, Writing – review & editing. RK: Data curation, Investigation, Supervision, Writing – review & editing. MT: Data curation, Investigation, Supervision, Writing – review & editing. HO: Data curation, Investigation, Supervision, Writing – review & editing. TYa: Conceptualization, Funding acquisition, Supervision, Writing – review & editing. YG: Conceptualization, Data curation, Funding acquisition, Investigation, Supervision, Writing – review & editing.
