# Supplementary material for: Association between loss of hypercoagulable phenotype, clinical features and complement pathway consumption in COVID-19
Source: Front Immunol. 2024 Mar 11;15:1337070. doi: 10.3389/fimmu.2024.1337070 (PMC10961343; doi:10.3389/fimmu.2024.1337070)
Supplement: Supplementary file 1 [file DataSheet_1.docx]

**ADDITIONAL FILE 1**

**Supplementary Methods**

**Data Collection and Management – Detailed Methods**

**Exclusion criteria of study participants**

The following patients were excluded from this study: 1) patients on home oxygen therapy, 2) patients with a confirmed diagnosis of a known coagulation disorder (protein C deficiency, hemophilia, etc.), 3) patients on oral anticoagulants (warfarin and DOAC) prior to admission, 4) patients with a baseline platelet count less than 100,000/µL, 5) patients with a confirmed diagnosis of hematologic cancer, 6) patients with “Do not intubate” orders, 7) patients from a hospital or nursing-home cluster of coronavirus disease 2019 (COVID-19). Patients who missed ROTEM evaluation due to cartridge shortage or evaluation failure were excluded from the analysis.

**Sample collection and processing**

For the ROTEM sigma^TM^ evaluation, blood sample collection using BD Vacutainer Citrate Tubes with 3.2% buffered sodium citrate solution were used within 4 h. For the analysis of the plasma viral load, BD Vacutainer Citrate Tubes were centrifuged at 1200 × g for 10 min and the plasma was recovered, and samples were stored at -80 °C until RNA extraction. Endotracheal aspirates were collected on admission, from mechanically ventilated patients diagnosed with COVID-19 and stored at -80 ℃ until use for whole genome sequencing of severe acute respiratory syndrome coronavirus 2 (SARS-CoV-2).

**Reverse-transcription polymerase chain reaction**

RNA extraction from plasma was performed according to the manufacturer’s protocol, using 140 μL of serum with QIAamp Viral RNA mini kit (QIAGEN, Germany), and RNA was used for reverse transcription by using cDNA synthesis kit (RiverTra ACE, TOYOBO, Japan). Reverse-transcription polymerase chain reaction (PCR) reactions were performed using the Mx3005p real-time PCR system (Agilent Technologies, USA) and the amplifications were performed using the SYBR green (THUNDERBIRD® Next SYBR® qPCR Mix, TOYOBO, Japan). The thermal cycling conditions were as follows: pre-denaturation step at 95 ºC for 10 min, followed by 40 cycles at 95 ºC for 30 s, 55 ºC for 60 s, and 72 ºC for 60 s. The experiments were carried out in duplicate for each data point. Human RNase P gene was used as an internal controller. The reverse and forward primer are shown in Table E2. Level of SARS-CoV-2 was recorded as the cycle threshold (Ct) value. The conversion of Ct value to viral copy number is based on a titration curve generated using synthetic partial viral RNA (RC351A, Takara-bio, JAPAN) with known copy number as a template for qPCR.

**Whole genome sequencing of SARS-CoV-2**

Tracheal aspirates samples with SARS-CoV-2 Ct values between 19 and 37 were selected for sequencing. Viral RNA was extracted using the QIAamp Viral RNA Mini Kit (Qiagen, Hilden, Germany), and libraries were constructed using the QIAseq SARS-CoV-2 Primer Panel and QIAseq FX Library Kit (Qiagen, Hilden, Germany) following the manufacturer's instructions. The libraries were indexed, pooled, and sequenced using the Illumina HiSeq X Ten (Macrogen Japan Corp., Tokyo, Japan). The sequence data was analyzed using the SARS-CoV-2 Workflows of CLC Genomic Workbench Ver.21.05 (CLC bio; Qiagen).

**Missing data**

Table E3 shows the completeness of the data for the overall cohort. Complete case analyses are used for all the tests.

**Figure E1:** Flow chart of patient inclusion (showing also reasons for exclusion of the 22 patients)

**Table E1.** Measurement methods of clinical laboratory parameters

| Laboratory parameters | Measurement methods/reagent |
| --- | --- |
| C3 | Turbidimetric immnoassay/N-Assey TIA C3-SH (Nittobo) |
| C4 | Turbidimetric immnoassay/N-Assey TIA C4-SH (Nittobo) |
| CH50 | Liposome immunoassay /Autokit CH50 Assay (FUJIFILM Wako Chemicals) |
| Fibrinogen | Clauss fibrinogen assay/Thrombocheck Fib(L) (Sysmex) |
| AT3 activity | Chromogenic amidolytic methods/ Rebohem AT (Sysmex) |
| PIC | Latex photometric immunoassay/ HISCL PIC reagent (Sysmex) |
| TAT | Chemiluminescent enzyme immunoassay / HISCL TAT reagent (Sysmex) |
| FDP | Latex immunoturbidimetry / Rias Auto P-FDP (Sysmex) |
| D-dimer | Latex immunoturbidimetry /Rias Auto D-dimer Neo (Sysmex) |
| KL6 | Chemiluminescent enzyme immunoassay /　Nanopia KL-6 Eisai (Eisai) |
| Total-Bilirubin | Chemical oxidation method / IATORO LQ T-BiL II (PHC) |
| vWF | fixed platelet aggregation method/ STA liatest vWF FR (DIAGNOSTICA STAGO) |
| sTM | Chemiluminescent enzyme immunoassay /Stacia CLEIA TM (LSI Medience) |
| PAI-1 | Latex photometric immunoassay / LPIA･tPAI test（LSI Medience） |

CH50 = total hemolytic complement; PIC = plasmin-alpha 2-plasmin inhibitor complex; TAT = thrombin-antithrombin complex; FDP = fibrin degradation product; KL-6 = Krebs von den lungen-6; vWF = von Willbrand factor; sTM = soluble thrombomodulin; PAI1 = plasminogen activator inhibitor-1.

**Table E2**. Primers for RT-qPCR

| **Primer** | **Sequence (5’→3’)** |
| --- | --- |
| CDC N1 Forward | GACCCCAAAATCAGCGAAAT |
| CDC N1 Reverse | TCTGGTTACTGCCAGTTGAATCTG |
| CDC N2 Forward | TTACAAACATTGGCCGCAAA |
| CDC N2 Reverse | GCGCGACATTCCGAAGAA |
| CDC RNase P Forward | AGATTTGGACCTGCGAGCG |
| CDC RNase P Reverse | GAGCGGCTGTCTCCACAAGT |

RT-qPCR = reverse-transcription quantitative polymerase chain reaction

**Table E3.** Comparison of baseline parameter between coagulation profiles in mechanically ventilated COVID-19

| **Variables** | **N^1^** | **Non-hypercoagulable, N=40**^2^ | **Hypercoagulable, N=44**^2^ | *P* value | Adjusted *P* value |
| --- | --- | --- | --- | --- | --- |
| Age (years) | 84 (100%) | 64 [56–73] | 60 [52– 69] | 0.149 | 0.455 |
| Tidal Volume, mL | 84 (100%) | 431 [367– 500] | 403 [347–459] | 0.182 | 0.455 |
| PEEP, mmH_2_O | 84 (100%) | 10 [10–12] | 11 [10–12] | 0.072 | 0.309 |
| PaO_2_-FiO_2_ ratio | 84 (100%) | 171 [123–203] | 168 [133–229] | 0.619 | 0.774 |
| Driving Pressure, mmH_2_O | 84 (100%) | 12 [10–14] | 12 [10–14] | 0.504 | 0.756 |
| AaDO_2_, mmHg | 84 (100%) | 249 [175–316] | 226 [171–292] | 0.395 | 0.741 |
| pH | 81 (96%) | 7.40 [7.37–7.45] | 7.38 [7.33–7.41] | 0.048 | 0.245 |
| Hematocrit, % | 79 (94%) | 39 [37–42] | 39 [36–42] | 0.352 | 0.704 |
| Creatinine, mg/dL | 84 (100%) | 0.84 [0.67–1.29] | 0.82 [0.67–1.13] | 0.684 | 0.811 |
| Total-bilrubin, mg/dL | 82 (98%) | 0.6 [0.5–0.7] | 0.55 [0.4–0.7] | 0.603 | 0.774 |
| Troponin-T, ng/mL | 67 (80%) | 0.01 [0.01–0.03] | 0.01 [0.01–0.02] | 0.175 | 0.455 |
| BNP, pg/mL | 69 (82%) | 33.4 [16.6–89.2] | 24.20 [15.45–47.1] | 0.171 | 0.455 |
| CRP, mg/dL | 84 (100%) | 5.96 [2.38–9.51] | 8.18 [4.32–11.91] | 0.2 | 0.462 |
| Ferritin, ng/mL | 75 (89%) | 1043 [643–1915] | 1138 [723–2193] | 0.468 | 0.756 |
| Procalcitonin, ng/mL | 75 (89%) | 0.1 [0.1, 0.2] | 0.1 [0.1–0.3] | 0.482 | 0.756 |
| KL6, U/mL | 80 (95%) | 461 [293–557] | 311 [260–447] | 0.11 | 0.413 |
| C3, mg/dL | 83 (99%) | 96 [73–118] | 116 [100–128] | 0.005 | 0.05 |
| C4, mg/dL | 83 (99%) | 31 [20, 42] | 31 [21–37] | 0.535 | 0.764 |
| CH50, U/mL | 73 (87%) | 62 [39–80] | 65 [49–78] | 0.703 | 0.811 |
| Platelet count, ×10^3^/μL | 84 (100%) | 154 [126–204] | 246 [194–308] | <0.001 | <0.001 |
| Fibrinogen, mg/dL | 79 (94%) | 428 [359–534] | 551 [481–579] | 0.002 | 0.03 |
| AT3, % | 84 (100%) | 87 [70–95] | 92 [83–104] | 0.018 | 0.135 |
| D-Dimer, μg/mL | 84 (100%) | 1.85 [0.9–5.93] | 1.58 [1–4.7] | 0.597 | 0.774 |
| FDP, μg/mL | 82 (98%) | 4.1 [2.5–17.1] | 3.9 [2.7–8.85] | 0.83 | 0.859 |
| TAT, ng/mL | 84 (100%) | 5.85 [3.53–13.32] | 5.95 [3.7–10.68] | 0.774 | 0.829 |
| PIC, μg/mL | 84 (100%) | 1.69 [1.28–2.41] | 1.79 [1.41–2.31] | 0.964 | 0.964 |
| LAC | 72 (86%) | 1.25 [1.10–1.40] | 1.35 [1.20–1.50] | 0.049 | 0.245 |
| PAI1, ng/mL | 72 (86%) | 28 [15.8–53] | 31.5 [14.5–55] | 0.756 | 0.829 |
| sTM, U/mL | 77 (92%) | 20.5 [14.4–37.3] | 24 [15.2–50] | 0.497 | 0.756 |
| vWF, % | 72 (86%) | 260 [226–298] | 235 [198–290] | 0.259 | 0.555 |

*P* values were adjusted using Benjamini–Hochberg procedure to decrease type I error of multiple comparison. ^1^shows numbers (proportions) of each variable without missing. ^2^ median (IQR). BNP = brain natriuretic peptide; CRP = C reactive protein; KL-6 = K den lungen-6 ; CH50 = total hemolytic complement; AT3 = antithrombin 3; FDP = fibrin degradation product; TAT = thrombin-antithrombin complex; PIC = plasmin-alpha 2-plasmin inhibitor complex ; PAI1 = plasminogen activator inhibitor-1; LAC = lupus anticoagulant; sTM = soluble thrombomodulin; vWF = von Willbrand factor.

**Table E4.** Proportion of data available for the analysis shown in Figure 6

|  | Day 0 | Day 1 | Day 2 | Day 3 | Day 4 | Day 5 | Day 6 |
| --- | --- | --- | --- | --- | --- | --- | --- |
| Total patients | 62 | 62 | 62 | 62 | 62 | 62 | 62 |
| C3 | 62 (100%) | 61 (98%) | 61 (98%) | 62 (100%) | 62 (100%) | 56 (90%) | 50 (81%) |
| C4 | 62 (100%) | 61 (98%) | 61 (98%) | 62 (100%) | 62 (100%) | 56 (90%) | 50 (81%) |
| CH50 | 62 (100%) | 60 (97%) | 61 (98%) | 62 (100%) | 62 (100%) | 56 (90%) | 50 (81%) |
| Platelet count | 62 (100%) | 62 (100%) | 62 (100%) | 62 (100%) | 62 (100%) | 56 (90%) | 50 (81%) |
| Fibrinogen | 62 (100%) | 62 (100%) | 62 (100%) | 62 (100%) | 62 (100%) | 56 (90%) | 50 (81%) |
| AT3 activity | 62 (100%) | 62 (100%) | 62 (100%) | 62 (100%) | 62 (100%) | 56 (90%) | 50 (81%) |
| PIC | 62 (100%) | 62 (100%) | 62 (100%) | 62 (100%) | 62 (100%) | 56 (90%) | 50 (81%) |
| TAT | 62 (100%) | 62 (100%) | 62 (100%) | 62 (100%) | 62 (100%) | 56 (90%) | 50 (81%) |
| FDP | 62 (100%) | 62 (100%) | 62 (100%) | 62 (100%) | 62 (100%) | 56 (90%) | 50 (81%) |
| D-dimer | 62 (100%) | 62 (100%) | 62 (100%) | 62 (100%) | 62 (100%) | 56 (90%) | 50 (81%) |
| Hematocrit | 62 (100%) | 62 (100%) | 62 (100%) | 62 (100%) | 62 (100%) | 56 (90%) | 50 (81%) |
| KL6 | 61 (98%) | 62 (100%) | 62 (100%) | 62 (100%) | 62 (100%) | 56 (90%) | 50 (81%) |
| Total-Bilirubin | 62 (100%) | 62 (100%) | 62 (100%) | 62 (100%) | 62 (100%) | 56 (90%) | 50 (81%) |
| vWF | 57 (92%) | 0 | 48 (77%) | 0 | 0 | 0 | 48 (77%) |
| sTM | 57 (92%) | 0 | 48 (77%) | 0 | 0 | 0 | 48 (77%) |
| PAI-1 | 57 (92%) | 0 | 48 (77%) | 0 | 0 | 0 | 48 (77%) |
| Viral Load | 52 (87%) | 0 | 0 | 0 | 0 | 0 | 0 |

TAT = thrombin-antithrombin complex; PIC = plasmin-alpha 2-plasmin inhibitor complex; FDP = fibrin degradation product; KL-6 = Krebs von den lungen-6; CH50 = total hemolytic complement; sTM = soluble thrombomodulin; vWF = von Willbrand factor; PAI1 = plasminogen activator inhibitor-1.
